# Innovation and Competency Development in Occupational Therapy Fieldwork During the COVID-19 Pandemic

**DOI:** 10.1177/00084174231190768

**Published:** 2023-08-03

**Authors:** Monique Gill, Anne Hunt, Andrea Duncan

**Keywords:** Education, Experiential learning, Competency development, Apprentissage par l’expérience, développement des compétences, éducation

## Abstract

**Background:** Occupational therapy clinical education was disrupted because of the COVID-19 pandemic. This introduced both challenges and opportunities in clinical fieldwork education and created a naturalistic opportunity to study the innovations that occurred. **Purpose:** To identify and describe fieldwork education innovations that occurred during the COVID-19 pandemic and understand how these clinical learning contexts impacted competency development in occupational therapy learners. **Method:** A qualitative multi-case study methodology was used. The participants (*N* = 28) were occupational therapy learners and preceptors who self-identified as having participated in an innovative fieldwork placement during the pandemic either as a preceptor or learner. Data were collected via in-depth interviews and analyzed to identify cases of innovation. Within and across case analyses were conducted to describe innovations and competencies addressed. **Findings:** Three cases of fieldwork innovations were identified: (a) Virtual Care; (b) Intrapreneurship; and (c) Administration. The commonly addressed competency domains across the cases were OT Expertise, Excellence in Practice, and Communication and Collaboration. The competency domain, culture, equity, and justice, was only addressed in the virtual care case. **Conclusion:** Our findings indicate that innovative fieldwork placements can support competency development in occupational therapy; however, this development is complex and contextually based.

## Résumé

**Description.** La formation clinique en ergothérapie a été perturbée par la pandémie de COVID-19. Cette situation a introduit à la fois des défis et des occasions à saisir dans la formation clinique et a créé une occasion naturelle d’étudier les innovations qui se sont produites. **But.** Identifier et décrire les innovations en matière de formation clinique qui ont eu lieu pendant la pandémie de COVID-19 et comprendre comment ces contextes d’apprentissage clinique ont affecté le développement des compétences des apprenants en ergothérapie. **Méthodologie.** Une étude qualitative multicas a été réalisée. Les participants (N = 28) étaient des précepteurs et des apprenants en ergothérapie qui ont déclaré avoir participé à ce titre à un stage pratique innovant pendant la pandémie. Les données ont été recueillies par le biais d’entretiens approfondis et analysées afin d’identifier les cas d’innovation. Des analyses intra et inter-cas ont été menées pour décrire les innovations et les compétences abordées. **Résultats.** Trois cas d’innovations ont été identifiés : 1) les soins virtuels; 2) l’intrapreneuriat; et 3) l’administration. Les domaines de compétences les plus fréquemment abordés dans ces cas étaient l’expertise en ergothérapie, l’excellence dans la pratique, ainsi que la communication et la collaboration. Le domaine de compétences « culture, équité et justice » n’a été abordé que dans le cas des soins virtuels. **Conclusion.** Nos résultats indiquent que les stages pratiques innovants peuvent favoriser le développement des compétences en ergothérapie; toutefois, ce développement est complexe et dépend du contexte.

The onset of the COVID-19 pandemic resulted in global challenges, as educational institutions needed to pivot to virtual learning (UNESCO, 2021). Specific to occupational therapy education programs, the pandemic introduced both significant challenges and opportunities in the clinical fieldwork education of occupational therapy learners.

Clinical education experiences, also known as fieldwork placements, are foundational within occupational therapy education (Robinson et al., 2012). Fieldwork placements are essential experiential learning that enables the learner to link theory and practice to develop essential competencies (ACOTRO et al., 2021; Casares et al., 2003). Occupational therapy (MScOT) learners are required to complete 1,000 hr of fieldwork to meet entry to practice qualifications (CAOT & ACOTUP, 2012).

Occupational therapy learners typically complete their fieldwork placements within traditional healthcare environments, such as hospitals, rehabilitation centers, or community settings, where learners are paired with a registered occupational therapist in an established role within these organizations. However, the occupational therapy profession also has a long-standing history of placing learners in role emerging placements, where they learn within programs or organizations where the occupational therapy role is new, less developed, or non-existent (Bossers et al., 1997). Role emerging placements have been found to have a positive impact on competency development and career trajectory for learners who completed these non-traditional fieldwork placements (Barker & Duncan, 2020; Syed & Duncan, 2019). More recently occupational therapy programs are adopting the term LEAP Placements (Leadership, Emerging, Advocacy, and Program Development) to categorize role emerging placements and other non-traditional fieldwork placements (Barker & Duncan, 2020).

The onset of the COVID-19 pandemic resulted in decreased availability of traditional fieldwork placements for occupational therapy learners, thus creating a significant void in the number of clinical experiential learning opportunities. To address this gap, university fieldwork educators engaged with clinical partners to develop innovative and creative approaches to provide clinical education to occupational therapy learners. Examples of these innovations included simulated patient experiences, virtual clinical experiences, project-based placements, and placements in non-traditional contexts. Anecdotally, both academic and clinical faculty have reported that both positive and less than desired outcomes resulted from these innovative placements. Despite this highly subjective hypothesis, we do not have a fulsome understanding of the innovations that have occurred, nor how these innovations impacted competency development amongst occupational therapy learners. Thus, the impact of these learning experiences remains unknown (MacKenzie et al., 2023). To our knowledge, there is no published peer-reviewed literature that explores these topics. Gustafsson (2020) cautions that we need to evaluate these pedagogies, which were designed to address a crisis, before we incorporate them into our curricula long term.

The global pandemic has forced health professional education programs to pivot in how they provide clinical learning, to ensure that health professional graduates are competent, system-ready, and able to meet the increasing demand for their services (Dedeilia et al., 2023; Gustafsson, 2020). The limitations on occupational therapy clinical education, resulting from the pandemic, created a naturalistic opportunity to understand pedagogical fieldwork innovation in a way it has not been studied previously. The objectives of this study are to (a) identify and describe clinical fieldwork education innovations that occurred as a result of the COVID-19 pandemic for MScOT learners within the University of Toronto catchment; and (b) understand how these clinical learning contexts impacted competency development in occupational therapy learners from the perspectives of fieldwork preceptors and learners themselves. The specific research question is “How do innovative clinical placement experiences implemented during the COVID-19 pandemic impact the development of required competencies in occupational therapy learners?”

## Methods

### Study Design

A qualitative multi-case study methodology (Yin, 1981) was used to address the research objectives and question. Case studies are most appropriate in real-life contexts that have unique historical significance and when researchers seek to study knowledge utilization when the phenomenon is inseparable from the context (Yin, 2012). In this study, exploring innovations and competency development in the context of clinical training, occurring during a global pandemic, involved a real-time context in which it is not possible to separate the innovations in clinical training from the global pandemic. In this study, the “case” represents a specific and unique clinical fieldwork educational innovation.

The research team's ontological and epistemological assumptions in designing this study were that (a) fieldwork innovations during the pandemic could be described and categorized into cases, (b) the participants involved in these innovations could reflect on how these influenced competency development, and (c) these competencies could be summarized and themes different from traditional fieldwork could be highlighted.

This study was approved by University of Toronto's Research Ethics Board (REB#40783).

### Study Population

This study sought experiences and perspectives of both clinical fieldwork preceptors and occupational therapy learners. The participants were occupational therapy learners and preceptors from the University of Toronto catchment area who identified as having participated in an innovative fieldwork placement, either as a preceptor or learner, during the pandemic from March 2020 to December 2020. A placement was concerned innovative if it did not fit the definition of a traditional fieldwork placement. Additional inclusion criteria for learners were that they had (a) successfully completed the MScOT program at the time of study enrollment and (b) been employed as an occupational therapist for a minimum of 3 months. The participants were excluded if they could not provide informed consent or reported that they did not feel they were able to speak to the competencies of an occupational therapist. The participants were recruited via an emailed study invitation to program graduates who had clinical placements during the pandemic and preceptors who hosted a placement during the pandemic from 2020 to 2021.

### Data Collection

Preceptors and learners who met the inclusion criteria were invited to participate in individual interviews, which occurred over zoom. A semi-structured interview guide facilitated the individual interviews and sought to elicit the participants’ experience with fieldwork innovation and perceptions of how this impacted competency development (Appendix A). All interviews were conducted by a trained research assistant who was not an occupational therapist and had no previous relationship with the learners or preceptors. This approach was consciously chosen in attempt to limit reporting bias, as most participants were previously known to members of the research team.

Interviews were recorded and transcribed verbatim. Informed consent was provided by all the study participants prior to participation.

### Data Analysis

All transcripts were reviewed independently by the research team and then entered into NVivo (QSR International, 1999) to prepare for analysis. The first step sought to identify the fieldwork innovations reported within the data. Using Yin's case study methodology, individual interviews were linked into case studies based on the specific fieldwork innovation(s). These case categorizations, and associated definitions, were discussed by the research team until consensus was reached.

Next, the research team coded each interview transcript deductively. Specifically, each interview was coded using the domains, competencies, and indicators from the *Competencies for Occupational Therapists in Canada* (ACOTRO et al., 2021) to identify competency development and/or gap among learners. How each domain was conceptualized by the research team, as it relates to this study, is outlined in Table 1. The coding was completed by two researchers, and the research team met to discuss discrepancies, which were discussed until consensus was reached. All interviews were grouped by “case,” and the data were then analyzed for themes *within* each of the case studies. This was completed by searching for, reviewing, and defining themes that were reflected by the responses from the participants (Braun & Clarke, 2006).

**Table 1:** Codebook Definitions Using Competencies for Occupational Therapists in Canada

Competency	Definition
Occupational Therapy (OT) Expertise	“Hard” or hands-on OT skills related to enabling occupation and the practice process: assessment, intervention, and evaluation skills. OTs analyze people's occupations and facilitate their participation, while collaborating with clients. This includes building of trust in a client–therapist relationship, analyzing the occupation in question while considering contextual factors, understanding the client's journey and aligning practice to their goals, assessing the situation, planning and implementing interventions, and clinical reasoning/thinking skills.
Excellence in Practice	Views OTs as lifelong learners, regularly self-evaluating and cognizant of external modifiers (emerging politics, technologies, etc.) to their practice, includes skills that are built throughout career as an OT including initiative taking, confidence, independence, leadership, problem-solving, creativity, flexibility, adaptability, advocacy for clients, and reflectivity
Communication and Collaboration	Communication: verbal and written communication, virtual communication, conflict resolution, connection/rapport building with colleagues and clients Collaboration: explaining the role of OTs to other professions, working with other professions/clients/stakeholders, setting boundaries and roles, conflict resolution, learning workplace social culture Does not include: independence/initiative (see excellence in practice), trust building in the practice process to achieve client goals (see OT expertise), and confidentiality (see professional responsibility)
Engagement with the Profession	Expand the profession through teaching, organizational/workplace/learner leadership, developing the practice/scope of OT, pushing boundaries of what OT is “known” to do, advocating for the use of OT outside traditional settings, changing practice and norms of practice/practice process, advocacy for change in practice Does not include: independence/initiative taking (see excellence in practice), independent leadership (see excellence in practice), advocacy for client (see excellence in practice)
Culture, Equity, and Justice	Learning about inequities and working toward inclusivity, acknowledge clients right to self-determination, challenging current under service of health resources across varying communities, cultural awareness and inclusion
Professional Responsibility	Adhering to legislations pertinent to service, and striving to mitigate risks, knowing what is within the OT scope and staying within it (i.e., not engaging in regulated activity), confidentiality and how to use technology to document/communicate

Lastly, consistent with Yin's Case Study methodology, the research team conducted a cross-case analysis. This process sought to identify common and differential “factors” across the multiple case studies represented within the data. Throughout this process, the researchers also noted how themes and factors may vary between the two participant groups (preceptors and learners).

Throughout the entire data analysis process, the research team met frequently to reflect, discuss, and challenge emerging assumptions and thematic summaries. The two co-PIs were conscious of potential researcher and confirmation biases and relied on the research assistants to guide the data analysis. To ensure dependability and credibility of the data, the members of the research team proposed alternate perspectives, highlighted flaws in data connections, and challenged assumptions before final conclusions were accepted.

## Findings

**Figure 1.**
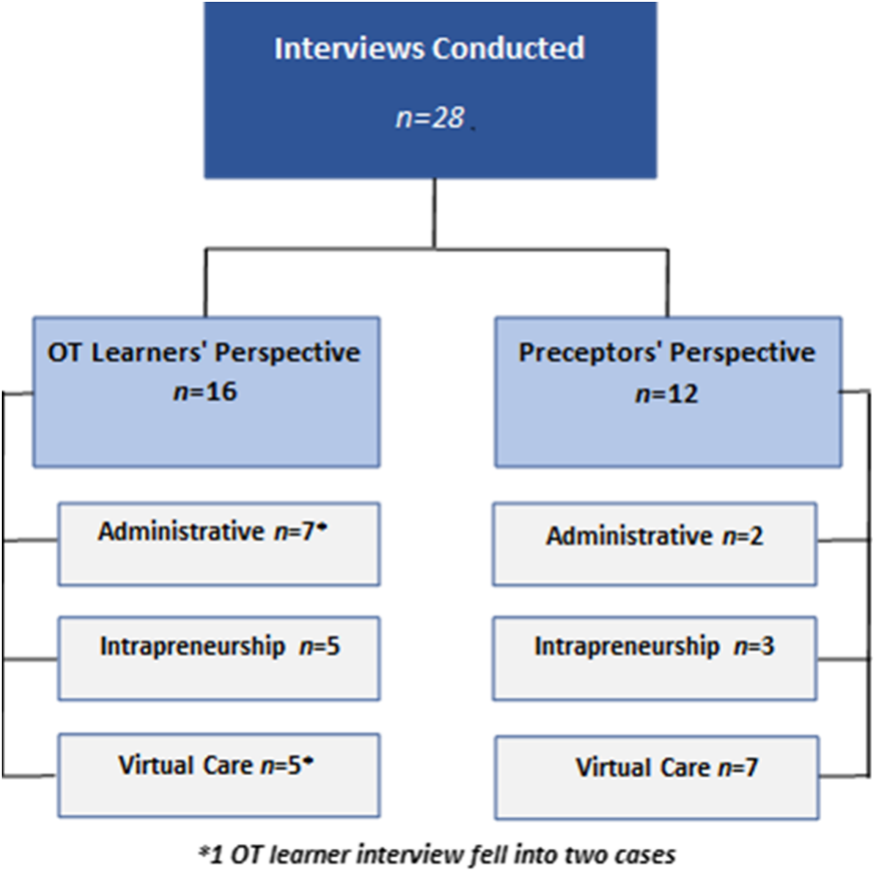
Cases of fieldwork innovation according to learners and preceptors.

In total, *N* = 28 participants engaged in the interviews. Of these, 16 participated from the perspective of the occupational therapy (OT) learners’ experience, and 12 represented the preceptors’ experience. No participant met the criteria for a dual role. We present our results by first describing the three case studies that identify the fieldwork innovations that occurred. Next, we describe the within case analysis that depicts what competencies were addressed in each case. Finally, we present the results across the identified cases, Figure 1 for Cases of Fieldwork Innovation according to Learners and Preceptors.

### Case Study Innovations

Three cases of fieldwork innovations were identified: (a) Administration, (b) Intrapreneurship, and (c) Virtual Care. Of the 28 interviews conducted, 11 were categorized as virtual care, eight as intrapreneurship, and eight as administration. One OT learner interview described fieldwork opportunities that fell into more than one category.

**Administration.** The interviews categorized under administration were unique in that they did not involve a clinical aspect or work with traditional occupational therapy “clients.” These fieldwork opportunities involved completing research projects, policy development, and program/guideline creation and evaluation. Learners completed these fieldwork opportunities within regulatory boards, educational settings, hospital settings, non-governmental organizations, non-for-profit organizations, community housing settings, and alongside management personnel in various positions.

**Intrapreneurship.** Intrapreneurship is defined as “entrepreneurial activity whereby organizations and organizational members take responsibility for creating innovation of any kind within an organization” (Rogelberg, 2017). Specifically, these cases encompassed fieldwork opportunities that advocated for occupational therapy services in new and non-traditional settings and included, but were not limited to, role emerging fieldwork opportunities. Innovations categorized as intrapreneurship included private practices taking on innovative approaches (e.g., art or nature-based approaches), not-for-profit organizations, public health settings, consultative roles, and community organizations. These fieldwork opportunities involved learners outlining the role of OT within the new setting, educating site staff regarding the role of OT, creating programs and educational material relevant to a non-traditional occupational therapy role, and carrying out innovative assessments or interventions with a wide range of clients. Examples of assessments and interventions during these fieldwork opportunities included the completion of population needs analyses, development of client education sessions and materials, and implementation of therapeutic interventions in non-traditional settings (e.g., during dance classes, in a camp setting).

**Virtual Care.** The interviews categorized under virtual care were identified as fieldwork opportunities within traditional in-person occupational therapy fieldwork settings that were transitioned to virtual care because of the COVID-19 pandemic. In this case, placements were completed in traditional clinical practice settings such as private practice, hospital settings, and international settings; however, traditional in-person care was shifted to virtual care. Learners completed traditional occupational therapy virtually, such as assessment and interventions with clients, documentation, and undertaking projects.

### Within Case Analysis—Competency Development

**Administration**. Within the administration cases, the top competencies highlighted by both preceptors and learners included the following: (a) applying the OT practice process in innovative ways (OT expertise); (b) critical thinking and clinical reasoning (OT expertise); (c) learning the role of OT in non-traditional settings (OT expertise); (d) the development of skills including independence, self-directedness, time management, and initiative (excellence in practice); and (e) expanding the OT practice into new settings or contexts (engagement with the profession). Skill development was also highlighted in the domains of communication/collaboration and professional responsibility; however, less emphasis on skill development was placed on these areas when compared to those listed prior.

Although administration placement cases involved conducting research projects, policy development, and program/guideline creation, preceptors and learners identified that the OT practice process was referred to and used frequently throughout placement developing skills within the occupational therapy expertise domain. The OT practice process is often used to guide clinical practice and facilitate change. However, throughout the interviews with preceptors and learners, it became clear that the OT practice process was used in innovative ways to inform research, policy, and program/guideline creation. An example of this is seen through the following quote from a learner.

“Facilitating change for the practice process… that was the whole placement… using the OT models and using the… connection between this setting and this program, what they wanted to achieve and what OT's… core values are and what we focus on, function and so on.” (Learner)

Administration placements cases also highlighted competency development in critical thinking, clinical reasoning, and learning the role of OT in non-traditional settings. Both competencies fall within the OT expertise domain. These assist learners in further developing essential skills needed for making decisions related to practice, as well as enabling occupation within their scope of practice in unfamiliar or challenging settings. Both competencies are necessary for the development of a comprehensive OT perspective/lens, where learners become proficient in grounding their practice in OT models of practice and frameworks.

“When I wrote that assistive device's resource, I… used my clinical reasoning to explain why this… particular object might be helpful for that population.” (Learner)

“I tried to really focus on the OT component, because that's why they’re here. Like, I want their takeaway to know that this is what occupational therapy is. Because here's an opportunity– like I feel like my role allows them to see it as… what OT really is and how it can be applied in different settings.” (Preceptor)

Finally, administrative cases highlighted major competency development in the domains of excellence in practice and engagement with the profession, specifically related to professional skill development and being an advocate within the profession. Additionally, preceptors and learners commonly identified skills that were noted to be developed throughout administration cases. These included independence, self-directedness, time management, and initiative. These skills highlight learner attributes that allowed them to work effectively in any setting they enter.

“Those life skills of just managing by yourself are … I think have been elevated through this pandemic… we might have lost some things: connection, in-person conversations, um, maybe some of the hands-on skills, but I think we’ve gained maybe more creative, insightful, innovative thinkers.” (Preceptor)

“There were no OTs at this placement. It was 95% online and a few things in-person here and there. Basically, I was working in the [program name] … to advocate for an OT role… what would occupational therapy look like here and how can an OT support? And at the end of the placement, I presented… and prepared a proposal for an OT role… it was great.” (Learner)

**Intrapreneurship.** Similarly in the intrapreneurship placement cases, the top competencies highlighted by both preceptors and learners included: (a) innovatively facilitating change through the OT practice process (OT expertise); (b) clinical reasoning (OT expertise); (c) client-centered practice (OT expertise); (d) practice knowledge (OT expertise); (e) innovative intervention skills (OT expertise); (f) expanding OT practice into new settings and contexts (engagement with the profession); and (g) skill development in independence, self-directedness, creativity, flexibility/adaptability, and problem-solving (excellence in practice). General communication/collaboration skill development was also discussed by learners and preceptors, however, often in collaboration with other competency and skill development. Learners identified competency development in the domains of culture, equity, and justice, as well as professional responsibility, as compared to preceptors who did not comment on these domains.

Intrapreneurship placement cases highlighted competency development in client-centered practice, practice knowledge, and innovative intervention skills within the OT expertise domain. As intrapreneurship cases involved role emerging opportunities, it is fitting that top competencies highlighted within OT expertise domain were focused on improving practice skills of the learners to address the challenging nature of working in an unfamiliar and innovative setting.

“We get to know the entire person… We wanna know about cognition, about, you know, their mental health. We wanna know about their physical health, their physical function, their social history, all that kind of stuff. And when we’re working towards… their next step, we have all of that in mind. And because our COVID patients couldn't have any visitors, and they were there for longer periods of time, ‘cause you have to be there at least two weeks, we just really had the opportunity to understand them as an entire being and not just a person in a bed who needs, you know, some functional mobility assessment or something like that.” (Learner)

“It was a big project plan to develop that [setting name] curriculum, something that we could do for a week at a time with a child, and she… mapped it all out. We created a curriculum, an activity binder of all sorts of activities, um, that we could be doing with the kids. The activities were slotted into domains of OT, so cognitive activities, physical activities, you know, social. Um, so she really was able to sort of see this whole process of developing something.” (Preceptor)

**Virtual Care.** Within the virtual care placement cases, the top competencies highlighted by both preceptors and learners included: (a) working through the OT practice process virtually (OT expertise); (b) skill development in work life balance, setting boundaries, problem-solving, independence, time management, and practice management (excellence in practice); (c) virtual communication and collaboration including written and verbal skills; (d) accessibility to resources (culture, equity, and justice); and (e) rules and regulations surrounding confidentiality in virtual spaces (professional responsibility). Neither learners nor preceptors identified competency development in the engagement with the profession domain.

Virtual care placement cases highlighted many similar skills developed in the previous cases. However, these cases also identified a unique niche set of skills specifically related to working in a virtual context. Learners and preceptors voiced that working through the OT practice process virtually built unique OT expertise, distinct communication and collaboration competencies, culture/equity/justice skills, and professional responsibility competencies regarding confidentiality in virtual spaces.

“…what was really interesting is with the virtual element, the OTs could no longer go in. So, what they were doing… a coaching model where instead, they were coaching the parents on how to do a lot of things… So, it actually really equipped the parents with skills that would be a million times more effective than an OT going in there and doing the skills.” (Learner)

“Collaboration and communication. I mean, those are fundamental to any role… I collaborated with my partner, in that we both were having to… split responsibilities and work together to ensure that we were having equal contributions to our work and meeting each other's strengths and weaknesses. And then also involves a lot of communication with my partner as well as [faculty member name] and [OT name] … to really make sure that we were meeting objectives… meeting deadlines.” (Learner)

“Resourcefulness, for sure, was a really big one because we had to– when I was in [country name], specifically, we had to work with what we had.” (Preceptor)

### Cross-Case Analysis—Competency Development Across Cases

When comparing cases, there were many similarities reported in the skills and competencies developed through innovative fieldwork placements. However, within each domain, different competencies were developed, dependent on the context of the case being discussed. This was seen in the domains of (a) OT expertise and (b) excellence in practice. Additionally, across cases, similar competencies were seen to be developed within the communication and collaboration domain with a few niche skills highlighted by learners and preceptors within virtual care cases.

**OT Expertise:** The OT expertise domain was addressed and developed in all cases. Within the OT expertise domain, competencies surrounding the OT practice process were commonly identified; however, these competencies were dependent on the case being discussed. Differences in the development of this competency can be seen in Table 2. In administration cases, learners applied the OT practice process to program development or resource development tasks in innovative ways. In intrapreneurship cases, learners learned to innovatively facilitate change with their clients. Finally, in virtual care placement cases, learners became familiar with applying the OT practice process in a virtual clinical setting.

**Table 2:** Examples of Competency Development Across Placement Cases

OT Expertise—OT Practice Process		
Administrative	Intrapreneurship	Virtual Care
“Facilitating change for the practice process… that was the whole placement… using the OT models and using the… connection between this setting and this program, what they wanted to achieve and what OT's… core values are and what we focus on, function and so on to… map it out and say, like… figuring out what are the additional things that OT can offer… to actually change the program and make it better.” (Learner applying the OT practice process).	“Our occupational therapy volunteers (learners) were surprised by how engaged and how beneficial it's been for the participant that they’re working with… they were amazed that… something that wasn’t pegged as a therapeutic class or a therapeutic session, um, is still giving a similar benefit.” (Preceptor on developing OT practice process skills on how to facilitate change in innovative ways among learners).	“When I was working one-on-one with clients at (private practice name), I definitely, like, was going through the thought process of like, “Okay. What interventions can I do with this client? What would benefit them? Why am I doing this?” And then sometimes during a session, maybe an activity that I had chosen wasn’t going well so I had to change things up during the session.” (Learner on using the OT practice process in a virtual care setting).
**Excellence in Practice**		
Administrative	Intrapreneurship	Virtual Care
“one thing that kind of stood out from… placement was more so the development of, like, project management, time management… just in a different way… you had so much freedom and to establish your own timelines and, um, what you were creating, I think that really puts a really huge emphasis on, you know, project management, time management, you know, allocating your own time, setting your own goals.” (Learner on independent goal setting and self-management of milestones and aims to allow for project completion).	“My creativity skills, my problem-solving skills… things that OTs honestly need on a daily basis ‘cause even meeting with clients now, like, it’s a lot of thinking on the spot. Like, as much as you come prepared, and… I always come prepared to see my clients… with ideas and… kind of a layout. But… you never know what they’re gonna say. So, it's always kind of, like, needing to be… really thinking actively and… being able to respond quickly… So, I was able from that placement to… build on my creativity skills” (Learner emphasizes creativity, adaptability, flexibility, and problem-solving skill development)	“You had to… problem-solve. And if you ran into an issue, you’d have to work through it… for the most part, you are on your own and you have to work through those problems… those are valuable skills to learn.” (Learner on development of problem-solving and independence skills as learners were required to practice through virtual settings, with emphasis on independent communication as preceptors were less available).

**Excellence in Practice:** The development of competencies within the excellence in practice domain also permeated through all placement cases. However, a similar pattern was seen in this domain, as different competencies were developed dependent on the context and demands of the placement case. Administration cases emphasized skills such as independence, self-directedness, time management, and initiative. During intrapreneurship cases of innovation, learners and preceptors voiced further development in skills such as independence, self-directedness, creativity, adaptability, and problem-solving. Finally, in virtual care cases, work life balance, problem-solving, independence, time management, creativity, and adaptability were highlighted as developed skills.

**Communication and Collaboration:** The development of competencies within the communication and collaboration domain was quite similar across all three cases. All fieldwork innovations required learners to develop competencies in communicating and collaborating effectively through virtual platforms whether written (e.g., through email, instant messaging, shared documents) or verbal through virtual/video conferencing (Zoom or Teams). However, there were a few niche skills highlighted by learners and preceptors as developed through virtual care placement cases due to the contextual nature of providing traditional care through a virtual platform. This included skills related to virtual group facilitation and virtual client communication (verbal and non-verbal).

“As you probably [are] aware, it's really hard to read people's body language… and… you get small little signals when you’re face-to-face with someone that you don’t get virtually… So, I think maybe with the… telerehab stuff, picking up on those subtle cues was a skill that you had to develop a little more… maybe some people who do want to work in telerehab, they’ll be more skilled… right off the get-go because of those experiences.” (Learner)

“I would be facilitating groups of—could be, like… 8 to 10 people where everyone's gonna try to talk at the same time, so, I was able to develop… the competency with communicator and being able to… moderate that group…” (Learner)

### Competencies Not Addressed Across Placement Cases

Culture, equity, and justice as a domain was not addressed in two out of the three placement cases. The preceptors in intrapreneurship placement cases and preceptors, as well as learners, in administration placement cases did not explicitly identify this as a competency that was developed.

## Discussion

This research study sought to identify and describe the academic fieldwork innovations that occurred within an occupational therapy clinical education program as a result of the COVID-19 pandemic. Specifically, we aimed to understand how these innovations influenced competency development in occupational therapy learners. Three cases of innovation were identified and included: Administration, Intrapreneurship, and Virtual Care. Differences and similarities were found across these innovations in relation to how occupational therapy competencies were developed. A major finding within this study included that regardless of the type of innovation, occupational therapy learners still developed competencies within the domains of occupational therapy expertise, excellence in practice, and communication and collaboration. However, subtle shifts in the types of competencies and skills that were developed within each innovative case were reported. These findings have promising implications for the future of occupational therapy curricula in Canada and internationally. As the profession increasingly embraces role emerging (Golos & Tekuzener, 2021) and LEAP (Barker & Duncan, 2020) fieldwork placements, the evidence from this study contributes to the conversation that these innovative placements do in fact develop competencies among occupational therapy learners.

The results of this study also highlighted findings related to the relationship between identified innovations and the current state of academic education. Additionally, there is an apparent lack of attention paid to competency development within the domain of culture, equity, and justice that is worthy of discussion.

### Clinical Fieldwork Innovations and Academic Education

The clinical fieldwork innovations identified in this study are very complementary to the academic education provided to learners pre-COVID-19 within the occupational therapy program at the University of Toronto. These identified innovations were not surprising to the research team as they were present pre-pandemic on a smaller scale during traditional and role emerging fieldwork placements. It is somewhat surprising nonetheless that learners and preceptors identified these as innovations resulting from the pandemic.

Most learners at University of Toronto complete three traditional fieldwork placements and one LEAP (Leadership, Emerging, Advocacy, and Program Development) (Barker & Duncan, 2020) fieldwork placement during their training. Pre-pandemic, both types of fieldwork placements commonly involved aspects of virtual care, intrapreneurship, and administration innovations. During the COVID-19 pandemic, fieldwork offers for these innovations were more plentiful and became more acceptable and valued by students and preceptors. For example, pre-pandemic, virtual care placements were less popular with students, and placement offers were rare. The pandemic resulted in occupational therapy practice shifts from in-person to virtual care and more placement offers in virtual care settings. Developments of competencies and skills that previously developed over the entire course of traditional placements were now being developed during more innovative placements. This includes skills within essential domains such as excellence in practice (e.g., leadership, initiative, creativity, problem-solving), communication and collaboration (e.g., virtual communication and collaboration skills), and engagement with the profession (e.g., learning to advocate and push the boundaries of OT practice).

### Culture, Equity, and Justice

The profession of occupational therapy has made a conscious effort to “promote equity in practice” and “promote anti-oppressive behaviour” (ACOTRO et al., 2021). While the culture, equity, and justice domain has only recently taken a more centralized focus in Canadian occupational therapy competencies, working toward inclusivity, and addressing inequities within OT practice, practice settings, and healthcare systems, has always been taught throughout OT curricula and explored with OT learners (Trentham et al., 2006). Specific to the University of Toronto's program, topics surrounding culture, equity, and justice are interwoven within OT practice courses across the life span, ranging from the enablement of occupation among the pediatric population into courses addressing topics related to older adults. This construct also appears in research and mentorship courses. Despite this emphasis in the program, the study participants did not explicitly mention this domain or the associated competencies when discussing the fieldwork innovations.

Despite this gap, in the virtual cases, there was reference to access to care and resources. We hypothesize some reasons for this. Both learners and preceptors highlighted that virtual care meant that technology was required to provide services. Virtual care meant that service could be provided to clients in underserved areas/rural areas and resulted in international virtual fieldwork placements. Learners and preceptors working together to provide services within these remote or international environments may have been more likely to voice differences in cultural norms and practices as the primary focus of these cases was to provide direct OT services to clients through virtual means. We postulate that the different foci in administrative and intrapreneurship cases resulted in differences in competency development across cases. Occupational therapy educators may wish to attend to the foci of placement innovations to ensure breadth of competency development in learners.

Within the newly developed Competencies for Occupational Therapists in Canada (2021), culture, equity, and justice has been identified as a standalone competency domain, independent of others. It is possible that explicitly addressing culture, equity, and justice is novel for preceptors and learners. Learners may begin to explicitly acknowledge their need to address this area within their future practice. This transition toward an explicit identification of culture, equity, and justice, as a competency domain, may lead to learners and preceptors identifying skill development and creating learner goals specifically within this area. As a future direction, reproduction of this study may lead to interesting results as vocalized skills among the culture, equity, and justice competency among innovations may become more prevalent in time among both learners and preceptors.

### Future Directions

While this study found that occupational therapy competencies can be developed within innovative fieldwork placements, there remains more to be studied in relation to fieldwork innovations and competency development in OT learners following the COVID-19 pandemic. Interview data did not completely fit neatly into each case and were identified by the research team as requiring further analysis. This data raised many questions and future research should explore the following: (a) the apparent link between confidence and competence among learners; (b) learners’ perceptions of their expected performance and its relation to their actual performance on fieldwork placement; (c) the impact of rapport and relationship building between learners and preceptors on learners’ competency and skill development; (d) exploration of how to educate both preceptors and learners on the idea that competency development does indeed occur in innovative fieldwork placements to ensure that these placements are valued as important learning experiences in fieldwork education; and (e) a potential reproduction of this study in the coming years as the newer domains within the COTC become more widely familiar among OT learners and preceptors.

### Limitations

There were limitations to our study. Reporting and response bias may have been prevalent as the Co-Principal Investigators were well known to learners and preceptors and there may have been some inherent conflict of interest. Additionally, the PIs pre-conceived hypotheses may have influenced the data analysis. To mitigate these risks, a research assistant who was not an occupational therapist conducted all the interviews, and de-identified interview transcripts prior to data analysis.

Additionally, the COTC document was introduced in 2021, in the midst of this study, and preceptors and students may not have been familiar with the newer language, domains, and indicators within the document. While there are similarities between the old and the new competencies, there are many differences as well (e.g., explicitly naming culture, equity, and justice as a domain). This may have impacted how the participants discussed competencies and their development.

## Conclusion

Our findings indicate that innovative fieldwork placements that occurred during the COVID-19 pandemic (administration, intrapreneurship, and virtual care) did support competency development in occupational therapy; however, this development is complex and contextually based. Common competencies addressed included occupational therapy expertise, excellence in practice, and communication and collaboration. Additional research is needed to understand competency development in relation to culture, equity, and justice, which was not commonly addressed across cases of innovation in our study.

### Key Messages

- Fieldwork innovations at the University of Toronto that occurred during the COVID-19 pandemic can be categorized into administration, intrapreneurship, and virtual care cases.
- All these fieldwork innovations continued to develop competencies among occupational therapy learners, especially within the domains of occupational therapy expertise, excellence in practice, and communication and collaboration.
- When discussing competency development of occupational therapy learners within the context of fieldwork innovation, learners and preceptors are largely silent on the domain of culture, equity, and justice.

## Appendix A—Semi-Structured Interview Guides

1) Are you responding from a student-learner role, or from a preceptor perspective?

### MScOT Student Interview Guide

1. Can you tell me a little bit about yourself?
  - When did you graduate? (if recent, congratulations) Aug 2020
  - Are you working now? If yes, where? What is your role there?
2. What was your experience with clinical fieldwork placements during your OT schooling?
3. Can I get to classify your placements based on the degree of innovation vs traditional setting?
  - [If asked about what we mean by “innovation”:] I leave it up to you to define innovation. Can be either virtual or in person. Something that was novel compared to your traditional expectations of a fieldwork placement in OT.
4. What was the project, what was the innovation, what was unique about it.
  - Where did you complete your placements?
  - What was your role? What were the clinical and / or project activities?
  - What was the structure of your supervision / preceptors?
5. In what ways did you find yourself challenged, either in a positive or negative way?
6. Thinking about competencies, what do you think are some of the competencies or skills that are?
  - most developed in an innovative setting?
  - Least?
  - Unchanged either way?
7. Thinking about competencies, what do you think are some of the competencies or skills that are?
  - most developed in a traditional setting
  - Least?
  - Unchanged either way?
8. How well do you think the fieldwork placements align with your career goals?
9. If you had a magic wand, what would you change about your fieldwork placements in retrospect?
  - Anything, including structure, level of supervision, your role, etc.
10. Thank you for your honesty, time and vulnerability in reflecting on your experiences. Is there anything else you would like to share with about fieldwork innovations during a global pandemic and its impact on student competencies?

### Preceptor Interview Guide

1. Can you tell me about yourself and your role?
  - What is your job and where do you work?
2. Please share your previous experiences with offering occupational therapy student fieldwork placements.
  - How many placements have you supervised in the last 5 years?
  - Can you tell me about your specific role as a preceptor?
  - How do you usually like to support student learning during a fieldwork placement?
3. Okay, great. Now I’d like to understand your views on how fieldwork placements *in general* develop competencies of MScOT students. Can you please share your view on this?
  - What competencies are most developed through clinical FW experiences?
  - What competencies are least developed?
4. During the COVID-19 pandemic, quite a few sites, organizations and preceptors offered innovative type fieldwork learning opportunities to continue to support MScOT learners. Can you please share *your* experiences and / or particular innovations that you were part of?
  - What was particularly innovative or novel about your offer (this is key to understanding “the case”).
  - How was this innovation different from usual practice?
5. I’m really interested in your perspectives on how these unique and innovative fieldwork experiences impacted competency development of our MScOT learners? For instance,
  - Did these innovations enhance any particular competencies?
  - Did these innovations hinder any competencies development?
  - Are there some competencies that were not impacted either way?
6. Is there anything else you would like to share with about fieldwork innovations during a global pandemic and its impact on student competencies?
  - Thank you for your time.
